# DLL3 expression is a predictive marker of sensitivity to adjuvant chemotherapy for pulmonary LCNEC

**DOI:** 10.1111/1759-7714.13574

**Published:** 2020-07-21

**Authors:** Hiroyuki Ogawa, Yasuhiro Sakai, Wataru Nishio, Yusuke Fujibayashi, Megumi Nishikubo, Yuki Nishioka, Shinya Tane, Yoshitaka Kitamura, Tamotsu Sudo, Toshiko Sakuma, Masahiro Yoshimura

**Affiliations:** ^1^ Department of Thoracic Surgery Hyogo Cancer Center Akashi Japan; ^2^ Department of Pathology Hyogo Cancer Center Akashi Japan; ^3^ Section of Translational Research Hyogo Cancer Center Akashi Japan

**Keywords:** Adjuvant chemotherapy, delta‐like 3, large cell neuroendocrine carcinoma

## Abstract

**Background:**

The mammalian Notch family ligands delta‐like 3 (DLL3) is reported to be a potential therapeutic target for large cell neuroendocrine carcinomas (LCNEC). The effect of DLL3 expression on LCNEC prognosis has not yet been elucidated.

**Methods:**

We reviewed the medical records of 70 LCNEC patients undergoing surgical resection between 2001 and 2015 using a prospectively maintained database. We performed immunohistochemistry for DLL3 and investigated the correlation between the sensitivity of LCNEC to platinum‐based adjuvant chemotherapy.

**Results:**

DLL3 expression was positive in 26 (37.1%) LCNEC patients. A total of 23 patients (32.9%) received platinum‐based adjuvant chemotherapy. Among patients with DLL3 expression‐positive tumors, no difference was found in the five‐year overall survival (OS) or recurrence‐free survival (RFS) between patients with and without adjuvant chemotherapy (surgery + chemotherapy vs. surgery alone, five‐year OS: 58.3% vs. 35.7% *P* = 0.36, five‐year RFS: 41.7% vs. 35.7% *P* = 0.74). In contrast, among patients with DLL3‐negative tumors, significantly greater five‐year OS and RFS rates were observed for patients with adjuvant chemotherapy than for those without it (surgery + chemotherapy vs. surgery alone: five‐year OS: 90.0% vs. 26.9% *P*<0.01, five‐year RFS: 80.0% vs. 21.7% *P* < 0.01). A multivariate analysis for the RFS revealed that adjuvant chemotherapy was a significant independent prognostic factor among patients with DLL3‐negative tumors (hazard ratio [HR]: 0.05, 95% confidence interval [CI]: 0.01–0.41, *P* < 0.01), although it was not a factor among patients with DLL3‐positive tumors (HR: 0.73, 95% CI: 0.23–2.27, *P* = 0.58).

**Conclusions:**

Our results revealed that DLL3 is a predictive marker of sensitivity to platinum‐based adjuvant chemotherapy for LCNEC.

**Key points:**

**Significant findings of the study:**

DLL3 was a predictive marker of sensitivity to platinum‐based adjuvant chemotherapy for LCNEC. Among patients with DLL3 expression‐negative LCNEC, platinum‐based adjuvant chemotherapy significantly improved the OS and RFS, although it did not do so among patients with DLL3 expression‐positive LCNEC.

**What this study adds:**

Our results suggest that DLL3 expression‐positive LCNEC may be better treated with other types of adjuvant chemotherapy, such as the anti‐DLL3 therapies if these effects are confirmed by ongoing clinical research.

## Graphical abstract

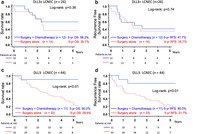

## Introduction

Large cell neuroendocrine carcinoma (LCNEC) is categorized as high‐grade neuroendocrine carcinoma (HGNEC) in the fourth edition of the World Health Organization Classification of Lung Tumors.^1^ The prognosis of LCNEC has been shown to be worse than that of other non‐small cell lung carcinomas (NSCLCs) because of their aggressive nature,^2^, ^3^ and several studies have shown that perioperative chemotherapy can remarkably improve the prognosis of LCNEC^4^, ^5^, ^6^, ^7^, ^8^, ^9^, ^10^, ^11^, ^12^, ^13^ patients.

Delta‐like protein 3 (DLL3) is an atypical member of the Notch receptor ligand family and reported to inhibit Notch signaling.^14^ There is growing evidence supporting a tumor‐suppressor role for Notch‐1 signaling in neuroendocrine tumors,^15^ and DLL3 is considered to promote neuroendocrine tumorigenesis by inhibiting the Notch receptor pathway.^16^ DLL3 is now considered a promising target of HGNEC, and DLL3‐targeting agents are in development.^17^, ^18^ George *et al*. reported that pulmonary LCNEC comprise two genomic subgroups with specific transcriptional patterns, defined as “type I LCNEC” and “type II LCNEC” in the first comprehensive molecular analysis of LCNEC.^1^ In that article, type I LCNEC showed higher expression of neuroendocrine genes, as well as of DLL3, and the downregulation of Notch pathway genes. In contrast, type II LCNEC showed a lower expression of neuroendocrine genes, including DLL3, and signs of Notch upregulation. Therefore, DLL3 expression may be a surrogate marker for distinguishing type I and II LCNEC.

These previous findings suggest that DLL3 expression will play a key role in clinical practice of LCNEC in the near future; however, the association of DLL3 expression with the clinicopathological features of LCNEC has not yet been elucidated. In the present study, we analyzed the effect of DLL3 expression on overall survival (OS) and recurrence free survival (RFS) in LCNEC and further analyzed the predictability of this expression for the efficacy of adjuvant chemotherapy.

## Methods

### Patient cohort

**Figure 1:**
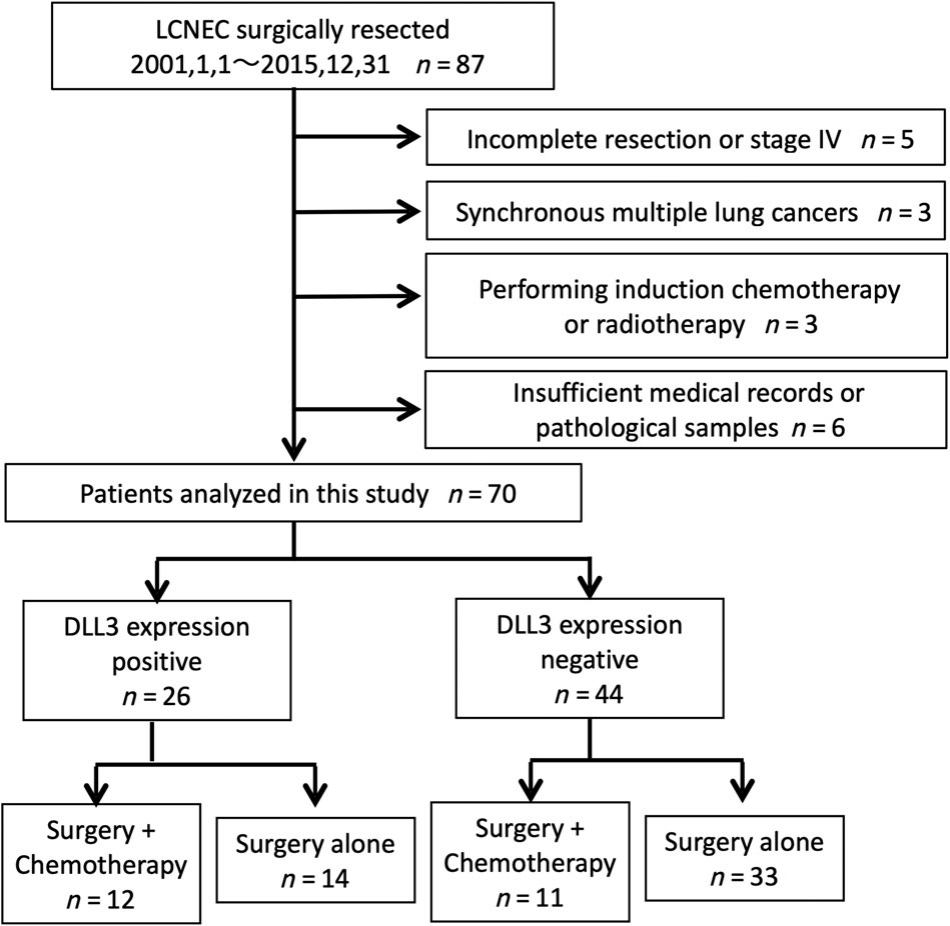
The patient population in this study.

We retrospectively reviewed a prospectively maintained clinical database of 87 pulmonary LCNEC patients who underwent surgical resection between 2001 and 2015. There were 17 patients who were excluded for the following reasons: pathological stage 4 or incomplete resection (*n* = 5), synchronous multiple lung cancers (*n* = 3), performing induction chemotherapy or radiotherapy (*n* = 3), and insufficient medical records or pathological samples (*n* = 6). The 70 remaining patients were analyzed (Fig 1).

Medical records provided information on the patients' age, sex, smoking status, lung function, surgical procedure, adjuvant chemotherapy, and pathological data. Determination of the disease stage was based on the seventh edition of TNM classification using the International Union Against Cancer (UICC) staging system.^19^ Surgery was performed for patients with an Eastern Cooperative Oncology Group Performance Status Scale (ECOG PS) of 0 or 1.^20^ The Hyogo Cancer Center Institutional Review Boards approved the study (NO. R666), and informed consent was obtained from all patients.

### Preoperative examination and follow‐up

Contrast‐enhanced chest and abdominal computed tomography (CT), positron emission tomography (PET)‐CT, and brain magnetic resonance imaging (MRI) were performed for preoperative staging. Patients were evaluated postoperatively at three‐month intervals for two years, at six‐month intervals for the subsequent three years, and annually thereafter. Follow‐up examinations included chest radiography, contrast‐enhanced CT, brain MRI, and bone scintigraphy as well as hematologic and biochemical analyses, including the measurement of the tumor markers. The OS and RFS were calculated according to the Kaplan‐Meier method, and the log‐rank test was used to evaluate differences in the distributions. OS was defined as the time interval between the date of surgery and the date of death. RFS was defined as the time interval between the date of surgery and the date of death without recurrence or the date of the first recurrence detected by a radiological examination.

### Histologic evaluation and immunohistochemistry

LCNEC was diagnosed based on the histopathological criteria described by the World Health Organization in 2015^2^: (i) neuroendocrine morphology such as an organoid, palisading, rosette‐like, or trabecular growth pattern; (ii) high mitotic count (≥11 per 10 high‐power fields [HPF]); (iii) tumor necrosis (often large zone); (iv) large cell size with a moderate amount of cytoplasm, vesicular or fine chromatin, and/or frequent nucleoli; and (v) positive immunostaining for one or more of the neuroendocrine markers synaptophysin, chromogranin A, and NCAM. In this study, we included both pure and combined LCNEC, in which at least one portion of neuroendocrine differentiation or morphology in NSCLC was LCNEC.

Immunohistochemical staining was performed on 4 μm thick formalin‐fixed, paraffin‐embedded sections. Sections were deparaffinized in xylene and rehydrated in graded alcohol baths. Antigen retrieval was performed by immersing the sections in 10 mM sodium citrate buffer (citric acid and sodium citrate, pH 6.0) for 20 minutes at 98°C in a water bath. Endogenous peroxidase activity was blocked in 3% hydrogen peroxide in water. Immunostaining was performed using the avidin–biotin complex technique (Dako LSAB2 System‐HRP kit; Dako, Carpinteria, CA, USA). Sections were incubated with anti‐DLL3 (monoclonal, clone E3J5R, CST, Boston, MA, USA) at a dilution of 1:300 at 4°C overnight and then incubated with biotinylated goat‐anti‐rabbit IgG, followed by peroxidase‐conjugated streptavidin complex. Complexes were visualized with 3,30‐diaminobenzidine, and the slides were counterstained with Mayer's hematoxylin, dehydrated and mounted. For the neuroendocrine markers, the sections were immunostained with the streptavidin‐biotin technique with an automated immunostainer (Benchmark; Ventana, Tucson, AZ, USA) according to the manufacturer's instructions. Antibodies against chromogranin A (polyclonal, 1:500 dilution; Dako, Glostrup, Denmark), synaptophysin (monoclonal, clone 27G12, 1:2 dilution; Nichirei, Tokyo, Japan), and CD56 (NCAM) (monoclonal, clone 1B6, 1:100 dilution; Novocastra, Newcastle, UK) were used.

**Figure 2:**
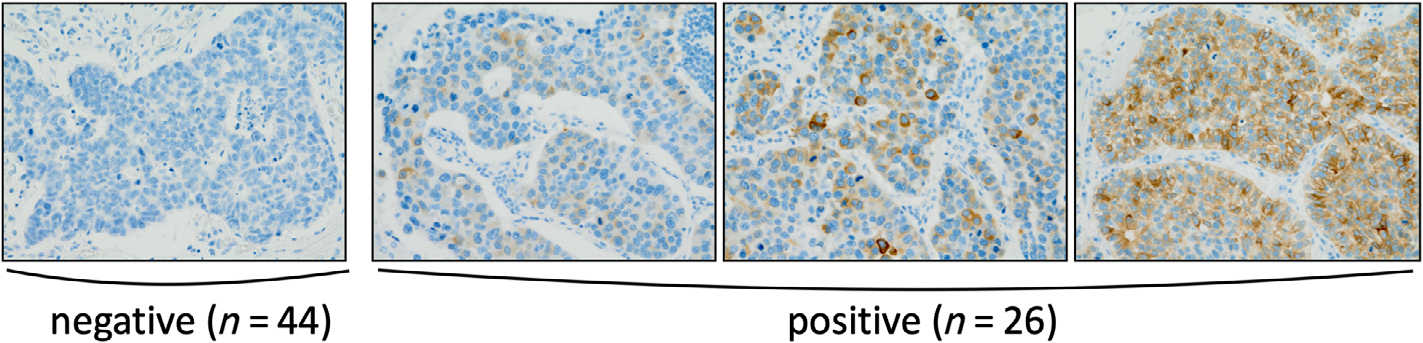
Immunohistochemistry of DLL3. The cutoff value was set at 1% for DLL3.

All samples were evaluated by an expert pathologist (Y.S.) without knowledge of the patient's outcome. In this study, the cutoff value for the percentage of positive tumor cells was set at 1% for DLL3 (Fig. 2). We set the cutoff value at 10% for chromogranin A, synaptophysin, and NCAM according to a previous study.^21^ We analyzed the predictability of DLL3 expression for the efficacy of adjuvant chemotherapy in the current study.

### Statistical analysis

Statistical analyses were carried out using the JMP 14 software program (SAS Institute, Cary, NC, USA). Student's *t*‐test and the chi‐square test were performed to assess the significance of the differences in age, sex, smoking status, lung function, surgical procedure, adjuvant chemotherapy, pathological stage (p‐stage) and the other pathological factors between the DLL3 expression‐positive and DLL3 expression‐negative groups. The OS and RFS were calculated according to the Kaplan‐Meier method, and differences in the distributions were evaluated by the log‐rank test. The Cox proportional hazards model was used to evaluate the association between prognostic factors and the RFS after pulmonary resection, with the hazards ratio (HR) and 95% confidence intervals (CIs). *P*‐values of <0.05 were considered statistically significant.

## Results

### Patient characteristics

The median follow‐up period of all patients was 37.8 months. DLL3 expression was positive in 26 (37.1%) LCNEC patients. Adjuvant chemotherapy was administered to 12 (46.2%) of the 26 patients in the DLL3 expression‐positive group, and 11 (25.0%) of the 44 patients in the DLL3 expression‐negative group. The patient characteristics are summarized in Table 1. The DLL3 expression‐positive LCNEC group included significantly younger patients (*P* < 0.01), patients with a better lung function (*P* < 0.01), and more patients with lymphatic permeation than the DLL3 expression‐negative group (*P* < 0.01). The five patients with combined LCNEC were all included in the DLL3 expression‐negative group. There was no marked difference in the sex, smoking status, surgical procedure, pathological stage, pathological tumor size, or rate of lymph node metastasis, pleural invasion, or vascular invasion between the patients with DLL3 expression‐positive and expression‐negative LCNEC. Patients who received adjuvant chemotherapy were significantly younger in both DLL3 expression positive and negative groups (*P* < 0.01). In DLL3 expression‐positive group, significantly more patients who underwent surgery alone were performed sublobar resection (*P* = 0.04). In the DLL3 expression‐negative group, significantly more patients with pleural invasion received adjuvant chemotherapy (*P* = 0.04).

**Table 1:** Characteristics of DLL3‐positive and DLL3‐negative pulmonary LCNEC patients

Factor	DLL3‐positive (*n* = 26)	DLL3‐negative (*n* = 44)	*P*‐value
Mean age, range (years)	65.6 (50–78)	71.1 (45–84)	**<0.01**
Sex			
Male	21 (81%)	39 (89%)	0.37
Female	5 (19%)	5 (11%)	
Smoking status			
Former or current	24 (92%)	41 (93%)	0.49
Never smoked	2 (8%)	3 (7%)	
Lung function			
Mean FEV1.0%	74.0%	66.6%	**<0.01**
Surgical procedure			
Lobectomy	22 (85%)	34 (77%)	0.45
Sublobar resection	4 (15%)	10 (23%)	
Adjuvant chemotherapy			
Performed	12 (46%)	11 (25%)	0.07
Pure or combined			
Pure LCNEC	26 (100%)	39 (89%)	0.07
Combined LCNEC	0 (0%)	5 (11%)	
Pathological stage			
IA	5 (19%)	16 (36%)	0.11
IB	10 (38%)	10 (22%)	
IIA	4 (15%)	5 (11%)	
IIB	0 (0%)	6 (14%)	
IIIA	6 (23%)	7 (16%)	
IIIB	1 (4%)	0 (0%)	
Pathological tumor size (mm)			
Mean	38.2	35.6	0.59
Lymph node metastasis			
pN 0	18 (69%)	33 (75%)	0.60
pN 1–2	8 (31%)	11 (25%)	
Pleural invasion			
(−)	14 (54%)	27 (61%)	0.54
(+)	12 (46%)	17 (39%)	
Vascular invasion			
(−)	5 (19%)	15 (34%)	0.18
(+)	21 (81%)	29 (66%)	
Lymphatic permeation			
(−)	4 (15%)	22 (50%)	**<0.01**
(+)	22 (85%)	22 (50%)	

### Adjuvant chemotherapy

The regimens of adjuvant chemotherapies are shown in Table 2. All patients received platinum‐based chemotherapy, and 18 patients (78.3%) received combination treatment with either irinotecan or etoposide. Five patients were administered tegafur‐uracil (UFT) orally after surgery and were included in the surgery group. One patient who received surgery alone and was DLL3 expression‐negative received postoperative radiation therapy to the margin of the tumor.

**Table 2:** Regimens of platinum‐based chemotherapy (*n* = 23)

Regimen	DLL3‐positive (*n* = 12)	DLL3‐negative (*n* = 11)
CDDP‐CPT‐11	8	5
CDDP + VP16	3	2
CDDP + VNR	0	1
CBDCA + VP16	0	1
CBDCA + PTX	1	2

CBDCA, carboplatin; CDDP, cisplatin; CPT‐11, irinotecan; PTX, paclitaxel; VP16, etoposide; VNR, vinorelbine.

### Correlation between DLL3 expression and neuroendocrine markers

We next examined the correlation between DLL3 expression and three neuroendocrine markers. The comparison of immunohistochemical staining for the three neuroendocrine markers between DLL3‐positive and DLL3‐negative LCNEC are summarized in Table 3. In accordance with a previous report,^1^ there was a strong correlation between DLL3 expression and expression of other neuroendocrine markers.

**Table 3:** Correlation between DLL3 expression and three neuroendocrine markers

Neuroendocrine marker	DLL3‐positive (*n* = 26)	DLL3‐negative (*n* = 44)	*P*‐value
Synaptophysin			
Positive	24 (92%)	23 (52%)	**<0.01**
Negative	2 (8%)	21 (48%)	
Chromogranin A			
Positive	23 (88%)	10 (23%)	**<0.01**
Negative	3 (12%)	34 (77%)	
NCAM			
Positive	25 (96%)	41 (93%)	0.60
Negative	1 (4%)	3 (7%)	
Mean number of positive neuroendocrine markers	2.77	1.68	**<0.01**

NCAM, neural cell adhesion molecule.

### Comparison of OS and RFS between DLL3‐positive and DLL3‐negative patients with and without adjuvant chemotherapy

**Figure 3:**
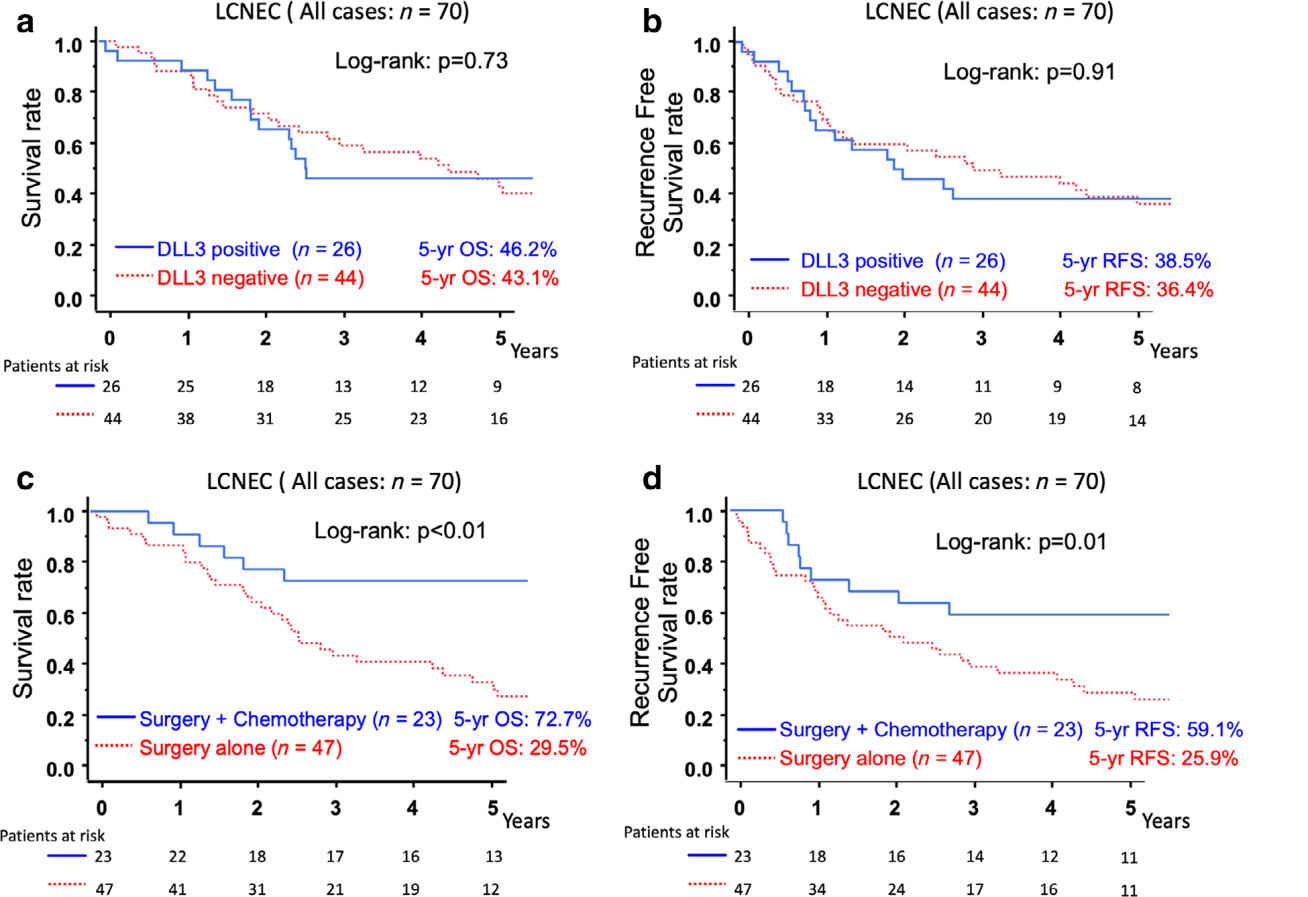
(**a**,**b**). Comparison of the overall survival (OS) (**a**) and recurrence‐free survival (RFS) (**b**) between DLL3 expression‐positive and expression‐negative LCNEC patients. (**c,d**). The comparison of the OS (**c**) and RFS (**d**) between LCNEC patients who underwent surgery with and without adjuvant chemotherapy. (**a**) (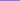) DLL3 positive (*n* = 26) five‐year OS: 46.2%, (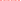) DLL3 negative (*n* = 44) five‐year OS: 43.1%; (**b**) (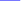) DLL3 positive (*n* = 26) five‐year RFS: 38.5%, (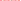) DLL3 negative (*n* = 44) five‐year RFS: 36.4%; (**c**) (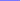) Surgery + chemotherapy (*n* = 23) five‐year OS: 72.7%, (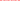) Surgery alone (*n* = 47) five‐year RFS: 29.5%; (**d**) (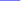) Surgery + chemotherapy (*n* = 23) five‐year RFS: 59.1%, (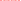) Surgery alone (*n* = 47) five‐year RFS: 29.5%.

We analyzed whether DLL3 expression was associated with the prognosis of LCNEC patients. There was no significant difference in the OS or RFS between the DLL3‐positive and DLL3‐negative patients (DLL3‐positive vs. DLL3‐negative, five‐year OS: 46.2% vs. 43.1% *P* = 0.73, five‐year RFS: 38.5% vs. 36.4% *P* = 0.91) (Fig. 3a,b). We then analyzed the efficacy of adjuvant chemotherapy in LCNEC patients. Adjuvant chemotherapy significantly improved the five‐year OS and RFS of LCNEC patients (surgery + chemotherapy vs. surgery alone: five‐year OS: 72.7% vs. 29.5% *P*<0.01, five‐year RFS: 59.1% vs. 25.9% *P* < 0.01) (Fig 3c,d).

**Figure 4:**
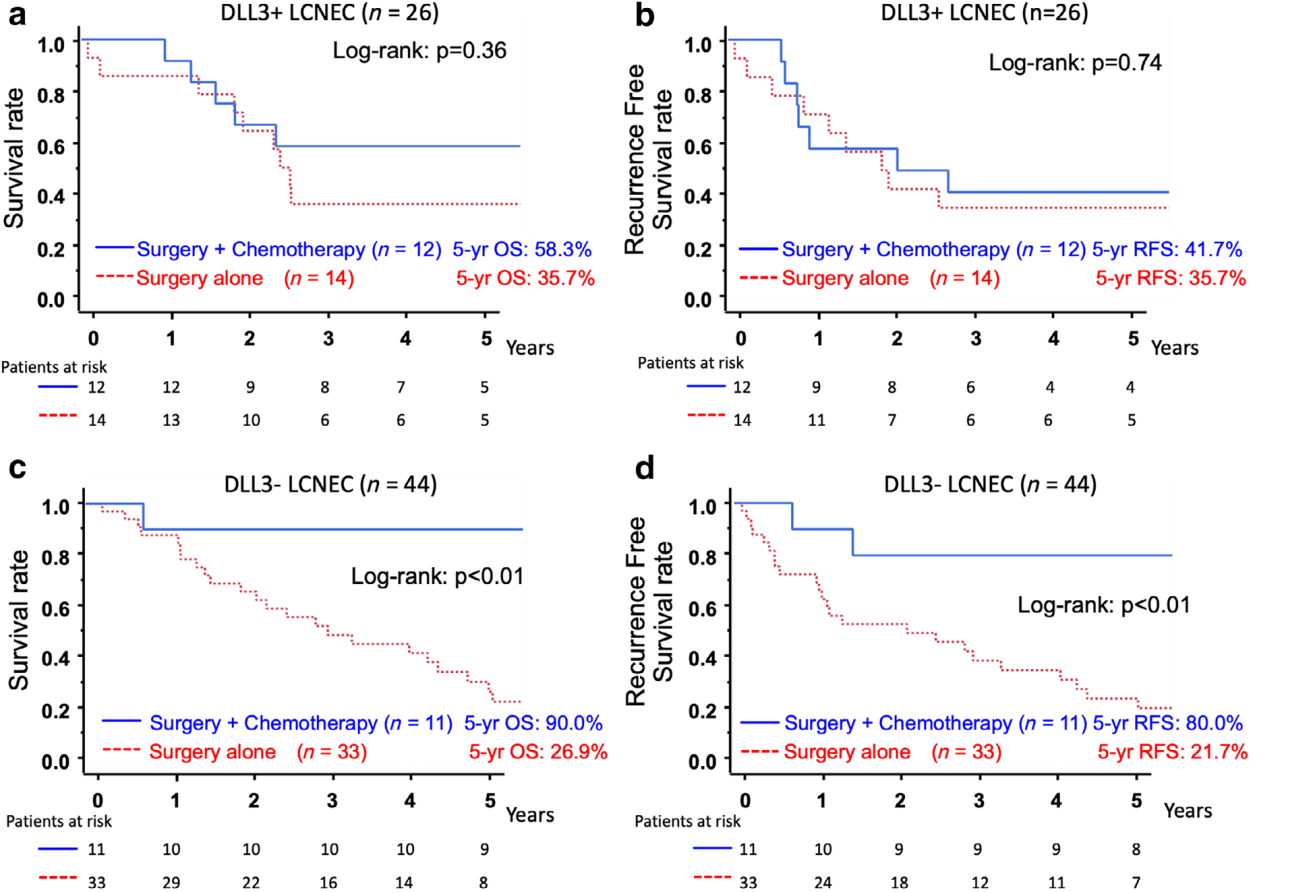
(**a**,**b**). The comparison of the overall survival (OS) (**a**) and recurrence‐free survival (RFS) (**b**) between DLL3 expression‐positive LCNEC patients who underwent surgery with and without adjuvant chemotherapy. (**c**,**d**). The comparison of the OS (**c**) and RFS (**d**) in DLL3 expression‐negative LCNEC patients who underwent surgery with and without adjuvant chemotherapy. (**a**) (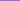) Surgery + chemotherapy (*n* = 12) five‐year OS: 58.3%, (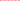) Surgery alone (*n* = 14) five‐year OS: 35.7%; (**b**) (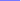) Surgery + chemotherapy (*n* = 12) five‐year RFS: 41.7%, (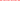) Surgery alone (*n* = 14) five‐year RFS: 35.7%; (**c**) (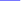) Surgery + chemotherapy (*n* = 11) five‐year OS: 90.0%, (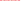) Surgery alone (*n* = 33) five‐year OS: 26.9%; (**d**) (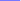) Surgery + chemotherapy (*n* = 11) five‐year RFS: 80.0%, (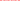) Surgery alone (*n* = 33) five‐year RFS: 21.7%.

Next, we evaluated whether DLL3 expression affects the sensitivity to adjuvant chemotherapy. Among patients with DLL3 expression‐positive LCNEC, no marked difference was found in the five‐year OS or RFS between patients with adjuvant chemotherapy and those without it (surgery + chemotherapy vs. surgery alone, five‐year OS: 58.3% vs. 35.7% *P* = 0.36, five‐year RFS: 41.7% vs. 35.7% *P* = 0.74) (Fig. 4a,b). In contrast, when the tumors were negative for DLL3, a significantly greater five‐year OS and RFS was observed for the patients with adjuvant chemotherapy than for those without it (surgery + chemotherapy vs. surgery alone: five‐year OS: 90.0% vs. 26.9% *P*<0.01, five‐year RFS: 80.0% vs. 21.7% *P* < 0.01) (Fig. 4c,d).

We performed a multivariate analysis for RFS to evaluate the efficacy of adjuvant chemotherapy among patients with DLL3 expression positive and negative LCNEC. We included three factors (pathological stage, surgical procedure, and adjuvant chemotherapy) that were considered to have an influence on the recurrence of patients with LCNEC.^7^, ^22^ A multivariate analysis for RFS revealed that adjuvant chemotherapy was a significant independent prognostic factor among patients with DLL3 expression‐negative LCNEC (HR: 0.05, 95% CI: 0.01–0.41, *P* < 0.01), although it was not a significant independent prognostic factor among patients with DLL3 expression‐positive LCNEC (HR: 0.73, 95% CI: 0.23–2.27, *P* = 0.58) (Table 4).

**Table 4:** Results of the multivariate analysis of prognostic factors for recurrence‐free survival

	DLL3‐positive			DLL3‐negative		
Variable	Hazard ratio	95% CI	*P*‐value	Hazard ratio	95% CI	*P*‐value
Pathological stage						
I	1.00			1.00		
II/III	2.66	0.84–9.20	0.10	2.25	0.86–5.81	0.10
Surgical procedure						
Lobectomy	1.00			1.00		
Sublobar resection	2.12	0.36–10.8	0.34	1.40	0.53–3.65	0.50
Adjuvant chemotherapy						
Not performed	1.00			1.00		
Performed	0.73	0.23–2.27	0.58	0.05	0.01–0.41	**<0.01**

## Discussion

In the present study, we demonstrated that DLL3 expression was a strong predictor of the efficacy of platinum‐based adjuvant chemotherapy for pulmonary LCNEC patients. Among patients with DLL3 expression‐negative LCNEC, platinum‐based adjuvant chemotherapy significantly improved the OS and RFS, although it did not do so among patients with DLL3 expression‐positive LCNEC. These results were confirmed by a multivariate analysis.

DLL3 is a unique Notch receptor ligand that inhibits the Notch pathway.^16^ DLL3 is normally expressed in fetal brain and plays a physiological role in development.^23^, ^24^, ^25^ Notch inactivation correlates to the neuroendocrine marker gene expression in LCNEC^1^ and small cell lung cancer (SCLC).^26^ DLL3 expression is closely related to neuroendocrine differentiation^15^ and is considered to promote neuroendocrine tumorigenesis in HGNEC.^16^ In addition, DLL3 is a novel target identified in tumor‐initiating cells isolated from SCLC and LCNEC patient‐derived xenografts.^16^ DLL3 is now a promising target of HGNEC, and DLL3 targeting agents are in development.^18^ Rovapituzumab tesirine (Rova‐T), a DLL3‐targeted antibody conjugate, showed encouraging single‐agent antitumor activity in patients with recurrent HGNEC in a phase 1 clinical trial.^17^ In this trial, DLL3 expression was a strong predictor of the efficacy of Rova‐T, and 35% of patients with DLL3 expression‐positive HGNEC had an objective response, whereas 0% of patients with DLL3 expression‐negative HGNEC had an objective response. After this trial, phase 2 clinical trial assessed safety and efficacy of Rova‐T for patients with DLL3 expression‐positive SCLC in the third‐line and beyond setting (TRINITY), and the results demonstrated modest clinical activity with associated toxicities.^27^ The anti‐DLL3/CD3 Bispecific T cell engager (BiTE) antibody (AMG 757) and a chimeric antigen receptor T cell therapy targeting DLL3 (AMG119) have been clinically evaluated in phase 1 clinical trials for the treatment of relapsed/refractory SCLC.^28^

There is increasing evidence that surgical resection alone is insufficient for treating LCNEC,^3^ and several researchers have reported that adjuvant platinum‐based chemotherapy for patients with LCNEC dramatically improved the surgical outcomes.^4^, ^5^, ^6^, ^7^, ^8^, ^9^, ^10^, ^11^, ^12^ While promising results of adjuvant chemotherapy for LCNEC have been reported, platinum‐based adjuvant chemotherapy did not improve the OS or RFS in patients with DLL3‐positive LCNEC in the present study. These results suggest that DLL3‐positive LCNEC may be better treated with other types of adjuvant therapy, such as anti‐DLL3 therapies if these effects are confirmed by ongoing clinical research. Once the findings in the present study are widely validated in further studies with larger sample sizes, the results will help form a rationale for conducting new clinical trials relevant to adjuvant anti‐DLL3 therapy for DLL3‐positive LCNEC.

We previously reported that the combination of three neuroendocrine markers was predictive of the sensitivity to platinum‐based chemotherapy.^8^ In the present study, we defined LCNEC that was positive for all three neuroendocrine markers (synaptophysin, chromogranin, and NCAM) as “triple‐positive LCNEC” and those that were negative for any of the markers as “nontriple‐positive LCNEC”. We revealed that adjuvant chemotherapy significantly improved the OS among patients with non‐triple‐positive LCNEC, although it did not improve the OS among those with triple‐positive LCNEC. Recently, George *et al*. revealed that LCNEC could be divided into two subtypes (type I and type II LCNEC) based on the genetic background according to a transcriptome analysis conducted with a next‐generation sequencer.^1^ We investigated the association between “triple‐positive LCNEC” and “non‐triple‐positive LCNEC” and “type I and type II LCNEC” based on the immunohistochemistry of DLL3, which is differentially expressed in type I and type II LCNEC. DLL3 expression‐positive LCNEC (type I LCNEC suspected) includes a large proportion of triple‐positive LCNEC, while DLL3 expression‐negative LCNEC (type II LCNEC suspected) mainly includes non‐triple‐positive LCNEC, suggesting a strong correlation between these two classifications. The present findings also imply that type II LCNEC might have greater sensitivity to platinum‐based adjuvant chemotherapy than type I LCNEC. In addition, the comparison of the clinicopathological characteristics between DLL3 expression‐positive and expression‐negative LCNEC revealed that DLL3 expression‐positive LCNEC induced lymphatic permeation at a significantly higher rate than DLL3 expression‐negative LCNEC. The invasion pattern might differ between DLL3 expression‐positive and expression‐negative LCNEC, which may reflect differences in the genetic background between type I and type II LCNEC.

The rate of DLL3 expression by immunohistochemistry was reported to be 65% among LCNEC patients,^16^ although it was only 37.1% in the current study. This discordance might be due to differences between the studies in the antibodies used. Brcic *et al*. compared the performance of four DLL3 antibodies in HGNEC samples and cell cultures and found poor results concerning the overall agreement.^29^ These findings suggest that we need take care concerning which antibody is used in order to ensure a proper understanding of the results and to maintain the reproducibility of studies relevant to DLL3 protein. The cutoff value of DLL3 expression is another important point to consider. We set the DLL3 expression cutoff value at 1% as the cutoff value of 1% was simple to use and could be used with high reproducibility, making it easily adaptable for future use in the clinical setting.

Several limitations associated with the present study warrant mention. First, this study was a retrospective, nonrandomized single‐center study with a small sample size. There may be some selection bias with regard to the performance of adjuvant chemotherapy, and its selection criteria have not been clarified. Second, the chemotherapy regimens were not standardized in this study. An NSCLC‐targeting regimen (platinum‐vinorelbine) was administered to patients with LCNEC before Rossi *et al*. reported that LCNEC responded better to an SCLC‐targeting regimen (platinum‐etoposide).^30^ However, despite the limitations of our study, we feel that our results are significant, and to our knowledge, this is the first report to demonstrate a relationship between DLL3 expression and the sensitivity of platinum‐based adjuvant chemotherapy. Further studies with larger sample sizes will be needed to verify the validity of our findings.

## Disclosure

There are no potential conflicts of interest.
